# Supplementary material for: A surge in food insecurity during the COVID-19 pandemic in a cohort in Mexico City
Source: PLoS One. 2024 May 10;19(5):e0297694. doi: 10.1371/journal.pone.0297694 (PMC11086887; doi:10.1371/journal.pone.0297694)
Supplement: Attachment — Submitted filename: Response to reviewers_15Oct2022.pdf [file pone.0297694.s002.pdf]

October 15th 2022

Courtney Elizabeth Heffernan
Academic Editor
PLOS ONE

Dear Academic editor.

On behalf of the authors of the original research article **"A surge in food insecurity during the COVID-19 pandemic in a cohort in Mexico City "**, we thank both the Editor and the reviewer´s on the thorough revision of our article. We have worked in the article to address all the comments and suggestions from the reviewers, and we are happy to submit a revised version of the manuscript and this rebuttal letter to respond to all comments and suggestions.

We look forward to the revision of this revised version and thank you again for your consideration and time dedicated to our article.

Sincerely,

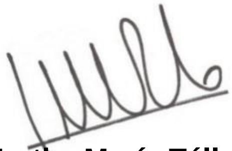

Martha María Téllez-Rojo, PhD
Corresponding Author

Reviewer #1: *I have published a number of studies regarding the measurement of food insecurity, and I feel like the authors did an adequate, even good, job. There was a clear objective, the data allow them to achieve their objective, and they used scientifically valid statistical methods. I appreciate the authors keeping the manuscript succinct. For example, they could have spent pages and pages discussing the ELEMENT cohort but they prudently remark instead that it is described elsewhere and they provide that citation.*

A couple of remarks:

1) *Use of the term "surge" in the title doesn't bother me but most reviewers do not like that kind of language.*

   Response: Our understanding is that the word "surge" is common in scientific literature. A quick search in PubMed yielded 19,623 results that use "surge" either in the title or abstract, and of those 2,869 use it in the title. If the reviewer does not mind, we would like to keep it as we think it is a good descriptor of the situation of food insecurity in the population "to rise suddenly to an excessive or abnormal value" (https://www.merriam-webster.com/dictionary/surge).

2) *On line 126, what is a "secular" fluctuation in food security? May be a translation issue here.*

   Response: We thank the reviewer for his/her comment. By secular we meant "long-term", the word is common in econometric literature, especially in time-series analyses. However, we agreed to change that term to "periodic", which we think is easier to understand. Line 154 "To evaluate periodic fluctuations in food insecurity during the study period…

3) *On lines 136 – 137 you remark that you only retain covariates that show statistical significance. In my field that is highly discouraged and is referred to as a form of "p hacking". People still do it all the time, you are just one of the few authors who admit it! If I were you though I would instead put your original estimations that also include the insignificant coefficients, lest someone use your paper as an example of what not to do. This isn't a huge deal for me but it is for some in my field.*

   Response: We thank the reviewer for this suggestion. We have removed said phrase from the statistical analysis sub-section and added the non-significant coefficients to table 4.

4) On Table 1 I don't understand what the COV% column means.

   Response: The COV% column describes the characteristics of the sample during the pandemic in visit COV. We have added the word "visit COV" in the description of the table.

Reviewer #2: *This study's goal is to estimate the impact of the COVID-19 pandemic on the occurrence of food insecurity in a cohort of women living in Mexico City, and the socioeconomic characteristics associated with food insecurity severity. I can definitely see this paper making contribution to still ongoing issue that countries face due to COVID so it is a very relevant topic. However, the manuscript could be stronger with a few suggestions listed below:*

Abstract

1) *Typo in the abstract, Results portion*

   Response: We have corrected the typo in the Results portion of the abstract.

Study population and sample.

2) *The timings of the visits are different across the years. Is that a concern? How can that impact results depending on what season of the year/crop cycle it is in?*

   Response: We thank the reviewer for his/her comments. We do agree that the time of year can have an effect on food insecurity due to potential yearly cyclical changes in the economy. Therefore, we deemed very important to control the model for the month of the year to control for this potential confounding effect.

3) *This should be written differently. "A total of 685 women had data on food security at least one data time point, while 432 women had information on all three time points."*

   Response: We have modified the writing to improve clarity: line 118-119 "432 women were interviewed on all three time points, and 685 women were interviewed at one time point at least."

Results

4) *Whereas all other types of food insecurity seem to have increased, it is puzzling that Mild food insecurity decreased. This is also mentioned the in abstract. More explanation and intuition here could be helpful.*

   Response: Overall food insecurity increased 12.2 and 7.6 percentage points at COV stage (53.8%) compared to previous stages: T1 (46.2%) and T2 (41.6%), respectively, which is a noteworthy result. But we intended to highlight the fact that the increase was concentrated mostly in the highest levels of food insecurity, as combined moderate-severe food insecurity increased 15.2 points from T2 (1.6%) to COV (16.8%) and12.8 points from T1 (4%) to COV stage (16.8%). Therefore, the decrease only in mild food insecurity is not a positive transition, since these results indicate that the sample population transited to the more serious levels of food insecurity in the COV stage. We agree that the reported result in the abstract can be unclear, therefore we modify the text for clarity: "Food insecurity (any level) increased from 41.6% duting the pre-pandemic period to 53.8% in the pandemic stage. This increase was higher in the combined severe-moderate food insecurity levels: from 1.6% pre-pandemic to 16.8% during the pandemic". Additionally, we included the percentages of "Food insecurity (any level)" in Table 2 so all the results are shown and can be easily interpreted.

Variables	T1<br>2015-2017<br>N=522	T2<br>2016-2019<br>N=519	COV<br>2020<br>N=590
	%	%	%
**No insecurity**	**53.8**	**58.4**	**46.2**

**Food insecurity (any level)**	**46.2**	**41.6**	**53.8**
Mild	42.2	40.0	37.0
Moderate	3.5	1.0	9.0
Severe	0.5	0.6	7.8

5) *Line 131: missing comma*

   Response: We have inserted the comma.

6) *the Table 1: since determinant seems to be pre and post pandemic timing, it would be better to present table 1 by the survey rounds, rather than quintiles (similar to table 2)*

   Response: We thank the reviewer for this comment. We have two main objectives for our analysis: i) assess changes in food security using pre and post pandemic information of the same sample of families, and ii) assess if these changes affect more economically disadvantaged, that is, if families with lower socioeconomic status were more exposed to experience food insecurity during the sanitary crisis. Our hypothesis behind this objective is that demographic and economic characteristics of more vulnerable families increase the likelihood of food insecurity. Table 1 has the intention to show the reader which characteristics are associated with more disadvantaged families who are more likely to experienced food insecurity. On the other hand, most of the variables shown in Table 1 are unlikely to have changed after the pandemic, showing pre and post pandemic characteristics will not offer a lot of variation in the case.

7) *Line 177: missing "the"*

   Response: We have inserted the missing article.

8) *Figure 1 does not appear where its placed.*

   Response: We have inserted Figure 1 in the document.

Econometric method

9) *Usually there are strong reasons behind why authors would choose to use FE versus RE models. In this paper, there is no justification or mention of why both are done? Also, there are no robustness checks of the models.*

   Response: We agree with the reviewer that the choice of statistical model is utterly important. Unfortunately there is no "Hausman-like" test for Ordered Logit Regression implemented in the statistical package to help us objectively decide for the fixed or random effects ordinal logistic regression models. We believe that our choice to present both is actually a kind of robustness check, with different types of models yielding very similar results. Although fixed-effects models are theoretically known to be consistent, and thus likely preferable, the advantage of also presenting random-effects models is that they allow for time-fixed regressors to be included in the model. We have included this rationale in the revised statistical analysis section. (line 167-170)

10) *Since the month of data collection is different, I thought I saw somewhere that authors control for that, however, I don't see that in Table 4. And how does controlling for months correlate with the survey rounds themselves? What is the correlation?*

Response: We apologize for our omission. The models did include months of the year as covariates, we now display the full set of coefficients in the revised Table 4. Months of the year were indeed significantly concordant with the timing of the surveys. The Spearman correlation of the month of the year with the timing of the survey rounds was 0.12 with a highly significant p-value (<0.001).

11) *The authors state that they used a host of characteristics but they only present the ones that are statistically significant. Are the rest of the variables not presented because authors are running out of space in the paper? Mention of these variables in table notes would at least be expected so readers can see it at a glance.*

Response: We apologize for this omission. Please see response to reviewer 1 and our revised table, which now explicitly includes all tested regressors.

Discussion

12) *"To our knowledge, this is the first study with panel data that estimated the magnitude of the COVID-19 impact on food insecurity in Mexico". I might be wrong but I thought that the World Bank did it for many countries across the world and that should have included Mexico.*

Response: The studies from the WB did include Mexico; however, these studies do not rely on longitudinal information that uses changes in food security using the same set of families as in our case. To our knowledge, our study is the only example from Mexico with panel information.

13) *Line 217: grammar LB*

Response: We have modified the writing to improve clarity: "Additional information on food insecurity in Mexico is available in the ENCOVID-19 survey (COVID-19 effects on the well-being of Mexican households survey), a nationally representative cross-sectional survey conducted monthly by telephone".

Line 267: grammar LB

14) **Response**: The indicated text changed in response to other comments. We hope the new text has also improved in clarity.

15) *Line 269: the definition should be written early on since the study is about food insecurity*

Response: We have placed the definition in the introduction.

Reviewer #3: *The manuscript addresses a relevant issue for which evidence already exists in Mexico and other countries. Still, it contributes from a methodological perspective by using data from a panel study and incorporating a longitudinal analysis. Nevertheless, the article presents*

several problems regarding the representativeness of its results, context, dialogue with specialized international literature, hypothesis, and the SES variable.

16) *Representativeness of results. The authors work with a cohort of women recruited from maternity hospitals in Mexico City (following the cited paper because it is not mentioned in this manuscript). How this cohort represents information about the general population? Why study this group could be interesting for understanding food insecurity? What are the limits of this study concerning this sample? The authors should explain more details about the recruitment, deep sample characteristics, and their limitations.*

Response: We appreciate the reviewer's question. First, we wholly agree with the reviewer in that the study sample is not representative of the Mexican or even Mexico City General Population, and this is acknowledged in the discussion section or the article. Although this could be seen a limitation, it is a limitation that is shared by most cohort studies. However, it should be noted that renowned epidemiologists have argued that representativeness is not only sometimes unnecessary, but it can even become a hurdle. (1) Others have pointed out that representativeness is necessary for obtaining good estimates of prevalence, but unneeded for analytical studies, in the sense that common objections to non-representative cohorts rarely cause bias beyond that which could be expected in representative samples. (2)

On the other hand, longitudinal (panel) data that originates from cohort studies has many advantages over the, often representative, data that is common in cross-sectional samples, of which we have cited several examples. It has been established that panel data, by blending the inter-individual differences and intra-individual dynamics can provide: 1. More accurate inference of model parameters; 2. Greater capacity for capturing the complexity of human behavior than a single cross-section or time series data. These include: 2.a Constructing and testing more complicated hypotheses; 2.b Controlling the impact of omitted variables; 2.c Uncovering dynamic relationships. 2.d Generating more accurate predictions for individual outcomes by pooling the data rather than generating predictions of individual outcomes using the data on the individual in question; amongst others. (3)In summary, we believe the strengths of the longitudinal data in terms of facilitating the finding of causal relationships, more than compensates for limitations in terms of representativeness of the sample.

1. Kenneth J Rothman, John EJ Gallacher, Elizabeth E Hatch, Why representativeness should be avoided, International Journal of Epidemiology, Volume 42, Issue 4, August 2013, Pages 1012–1014, https://doi.org/10.1093/ije/dys223

2. Lorenzo Richiardi, Costanza Pizzi, Neil Pearce, Commentary: Representativeness is usually not necessary and often should be avoided, International Journal of Epidemiology, Volume 42, Issue 4, August 2013, Pages 1018–1022, https://doi.org/10.1093/ije/dyt103

3. Hsiao, Cheng, Panel Data Analysis - Advantages and Challenges (May 10, 2006). IEPR Working Paper No. 06.49, Available at SSRN: https://ssrn.com/abstract=902657 or http://dx.doi.org/10.2139/ssrn.902657

17) *The limits of this research imply that the authors must moderate some statements. For example, the abstract mentions that food insecurity soared in Mexico City in 2020 (in general), but in the same abstract, they mention that they work with data from mid and lower mid to low SES in Mexico City. If their sample came from this group of women, they must be cautious with generalizations. LB/LT*

Response: We agree with this comment. We have modified the statement as follows: "Food insecurity soared in Mexico City households **in our sample population** in 2020, during the COVID-19 pandemic".

18) *Context. The authors should give information about the Mexican contexts to understand the results better. What is Mexico's long-term situation of food insecurity (and obesity)? Does Mexico is in a nutritional transition? How many people have formal and informal employment? How is this situation different or like other Latin American countries?*

Response: We thank the reviewer for the comment. We added contextual information on obesity trends in Mexico as well as labor information in the text. We have also explained further how disruptions in family labor and economic well-being affect food security in general, and in Mexico in particular. Although we do think that adding information from the Mexican context will help the reader to better grasp the results of the paper, the mechanisms that are behind the rise of food insecurity in our sample can be translated to other contexts, in Mexico and other countries, as shown in previous research. Gaytan-Rossi et al (2020), for example, use nationally representative data from Mexico to document food security during the pandemic. As they explained, a raise in food insecurity can be directly linked to the loss of labor (and income), particularly among families in the informal sector of the economy. Examples in the literature from other countries found similar results (see for example Enriquez and Goldstein; 2020 and Manfrinato CV et al; 2020). Similarly, disruptions in food systems that brought about a shortage of some foods have been liked to higher food prices globally. As a consequence, some research points to an increase in cheaper, high-calorie foods by families who experienced income losses during the pandemic, affecting in turn nutrition.

References:

Enriquez D & Goldstein A (2020) Covid-19's socio-economic impact on low-income benefit recipients: early evidence from tracking surveys. SocArXiv. Published online: 16 June 2020. doi: 10.31235/osf.io/hpqd5.

Manfrinato CV, Marino A, Conde VF et al. (2020) High prevalence of food insecurity, the adverse impact of COVID-19 in Brazilian favela. MedRxiv. Published online: 04 August 2020. doi: 10.1101/2020.07.31.20166157.

World Bank (2022). Food Security Update. Available in https://www.worldbank.org/en/topic/agriculture/brief/food-security-update. (Accessed October 2022).

19) *Dialogue with specialized literature. The reference to the literature in the introduction is weak. Later in the discussion section, the results are primarily confronted with the Mexican surveys. That implies several problems. First, it does not allow situated the food insecurity*

during COVID as a world phenomenon. Second, it does not give justification to justify the variables used in the models. For example, in the introduction, the authors mentioned in a very general way that various variables can affect food insecurity without specifying the variables used in their work. In fact, they include variables that are not possible to measure in the model type, such as political instability. The authors need to go deeper into the variables' justification to understand those included in the models. At the same time, it is necessary to differentiate between macro and micro factors (you are working with individual variables).

Response: We have modified the introduction (and other sections of the article to be consistent with changes in the introduction) to present the food insecurity framework related to its dimensions and levels. We expect that this modification can show clearly the rationale of our analyses that uses information only at the household/individual level, and therefore justify the use of the selected variable for the analyses.

20) *Hypothesis. The authors do not declare any hypothesis. Maybe that is a result of a weak literature review. To improve the text, the authors should elaborate hypotheses founded on literature. That action also will enhance the justification of the model.*

Response: We thank the reviewer for her/his comment. In the revised version, we now explicitly include our hypothesis: "In this work, we tested the hypothesis that the COVID-19 pandemic caused a sudden increase on food insecurity in Mexico City women in the 2020-2021 period". (line 106-107)

21) *SES variable. Why did the authors use this method to build SES and not another proxy for education level (usually correlated)? Authors include both in their model and household's head education level seems to perform better. Stratification variables have an extensive discussion in social sciences. Even in epidemiology, some studies use this kind of indicator based on house assets (authors cited references); social sciences question the quality of these variables. The problem is visible regarding the results of this study because this variable behaves strangely in its link with food insecurity (see quintiles 2 and 4).*

Response: Thank you for your comment. We included both SES and education for reasons that we described as follows. First, as the reviewer suggested, both variables are likely to be correlated, but they do not measure the same dimensions of economic well-being. Braveman et al. (2005), for example, showed that measures of education and income included in health studies are correlated but not strong enough to justify the use of one or the other. Moreover, the same authors revised several studies in the United States. They found that earnings and other SES measures usually vary across the same educational level, showing that educational attainment differs for families considered equally poor. A potential explanation of this variation is the effect of other factors, like race, which the authors find to explain earning differences (they find evidence, for example, that Mexican and American adults of similar educational attainment report lower incomes). In the case of Mexico, we expect a similar pattern given recent research that documents that skin color (a proxy of race) predicts economic achievement that education cannot indicate

entirely (Campos-Vazquez, R.M., Medina-Cortina, E.M. Skin Color and Social Mobility: Evidence From Mexico. Demography 56, 321–343 (2019). https://doi.org/10.1007/s13524-018-0734-z. Winch C. The economic aims of education. J Philos Educ. 2002;36:101-117. Reynolds JR, Ross CE. Social stratification and health: education's benefit beyond economic status and social origins. Soc Probl. 1998;45:221-247.). Second, education can also reflect other non-economic social characteristics like skills and knowledge with potential effects on different outcomes, including food security. This is another reason for including education and SES simultaneously in our models. And third, socioeconomic status, measured through an asset index, is better suited to explain the economic capacity of households to face economic shocks, like the economic downturn experienced during the sanitary crisis due to Covid19 compared with other SES proxies. Shocks affecting food insecurity, in particular, seem to be better captured by a wealth index than other measures like income or expenditures because they better describe vulnerability to food insecurity (see, for example, the discussion by Hjelm et al., 2016, with data from five countries).

22) *Otherwise, in the abstract, the authors mention that they work with mid and lower SES, but in their data, they built a wealth index, including high "wealthiness." How is this possible? That is not explained anywhere. Two solutions are possible. Use education level (if the authors do not have other indicators). Better explain the index regarding the sample and its limitations.*

Response: Thank you. We agree with the reviewer. We have adjusted the text to avoid confusion. In the abstract, we mentioned that women in the cohort were from mid and lower SES, whereas in the analysis, we have women across quintiles of SES. The reason for this apparent contradiction is the following:

i. In the abstract, we referred to the SES status of households in previous phases of the COVID data collection phase. Back then, the investigators used a different index than the one used for our analysis. We have clarified this in the text (lines 273-278).

ii. For our analysis, we estimated a new SES index because we expected that SES levels could have changed since 2018, when the old index was calculated. However, in contrast with the original SES variable, the new index provides a **RELATIVE** measure of SES of households in the analytical sample. This means that we can compare how poor or rich women are ***in relation*** only to other women in the sample, not to the population in Mexico City. For the purpose of our analysis, we believe this variable is better suited to assess the effect of the pandemic on food security for relatively more vulnerable households. As explained in the text, the new SES was estimated via principal component analysis (PCA) which is standard in the literature (see previous response to comment 21).

23) *Other comments: Perhaps the word quintile is not the best word to describe grouping SES because it leads erroneously to think of a continuous variable with income.*

Response: The wealth index we used in this analysis is continuous. See responses above.

24) *Perception of food consumption does not seem justified insofar as it may have a high correlation with the food security scale, generating possible problems in the model. I think the modeling is correct to have a sensitivity to seasonal aspects. Also, the work with longitudinal models.*

Response: Thank you. We agree with the reviewer. We removed this variable from Table 1. Note, however, that perception of food consumption was not included in the statistical models. We also added a note in Table 3 clarifying which variables are included as a controls in both specifications.

25) *It is not clear the inclusion of SES media in table 1.*

Response: Thank you, we agree, we removed the value of the mean in Table 1.

26) *Table 4 four does not contain all the reference categories listed, although it is possible to assume it.*

Response: Thank you, we added the references in Table 4.

***
