# Supplementary material for: A surge in food insecurity during the COVID-19 pandemic in a cohort in Mexico City
Source: PLoS One. 2024 May 10;19(5):e0297694. doi: 10.1371/journal.pone.0297694 (PMC11086887; doi:10.1371/journal.pone.0297694)
Supplement: Attachment — Submitted filename: Response to reviewers_10Feb2023.pdf [file pone.0297694.s004.pdf]

February 10th 2023

Courtney Elizabeth Heffernan
Academic Editor
PLOS ONE

Dear Academic editor.

On behalf of the authors of the original research article **"A surge in food insecurity during the COVID-19 pandemic in a cohort in Mexico City "**, we thank both the Editor and the reviewer´s on the thorough revision of our article. We have worked in the article to address all the comments and suggestions from the editor and reviewer #2, and we are happy to submit a revised version of the manuscript and this rebuttal letter to respond to all comments and suggestions. Since the reviewer´s comments were included directly in the manuscript, please note that al responses in this letter are the same as the responses included directly in the "track changes" version of the revised manuscript.

We look forward to the revision of this revised version and thank you again for your consideration and time dedicated to our article.

Sincerely,

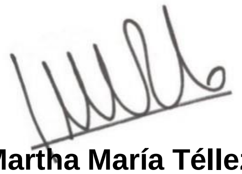

Martha María Téllez-Rojo, PhD
Corresponding Author

Editor comment: *Please review your reference list to ensure that it is complete and correct. If you have cited papers that have been retracted, please include the rationale for doing so in the manuscript text, or remove these references and replace them with relevant current references. Any changes to the reference list should be mentioned in the rebuttal letter that accompanies your revised manuscript. If you need to cite a retracted article, indicate the article's retracted status in the References list and also include a citation and full reference for the retraction notice.*
Response: We reviewed our reference list. We confirm that is correct and complete. We have not included any retracted articles. We added one new reference to respond a reviewer´s comment: 36. Castillo MJ, Galicia M, Castellano F. Evolución del costo de los alimentos ante el COVID-19. Chile: Centro Latinoamericano para el Desarrollo Rural, 2021 15.

Reviewer #2
Comment*: Replace with increased*
Response: We replaced the term "soared" with "increased", as suggested.

Comment*: The appropraite recommendation based on the study is to suggest that the government or ministry of agric provide some cushioning against food insecurity. This study did not focus on the effects of food security*
Response: Food security includes four dimensions: 1) food availability, 2) food access, 3) food utilization, and 4) stability. Alterations in any of the food security dimensions can result in a reduction of the quality and quantity of food consumed by household individuals. We would agree with the reviewer´s comment, if the insecurity problem observed in our sample population was due solely to food supply (food availability dimension), but this is not the case. By directly intervening in households, a program could help families with insecurity caused by disruption in all dimensions of insecurity: food availability, food access, food utilization, and stability. Based on this rationale, we propose to keep our original recommendation.

Comment: *Break into two sentences at least. This is too long*
Response: We edited the text to separate into two sentences, as suggested.

Comment*: This statement is not supported. Rephrase. Look at these papers*
Response: We rephrase the text as suggested.

Comment: *if there was a question about employment status during the covid lockdown it Will be Good to include it*
Response: Employment status was collected during the Covid stage with the question "whether the woman respondent had a job over the previous week", and results are included in Table 1. Nevertheless, the variable "having a job last week" was not included in the models as it is most likely an intermediate variable between the "Pandemic" variable and food insecurity. In other words, at least part of the effect of the pandemic on food insecurity is likely to be due to unemployment, therefore adjusting for said variable would cause an underestimation of the true effect. This information has been added to the updated "statistical analysis" sub-section.

Comment: *you did not present any framework*
Response: By conceptual framework we are referring to the background knowledge of food insecurity drivers that was included in the "Introduction". To clarify we edited the text accordingly.

Comment: *provide an explanation of why this was done*
Response: The reason for inclusion of the time variable (months of the year is described in the previous paragraph (lines 154-158). The rest of the covariates were deemed necessary to include as previous knowledge on food insecurity as per our literature review has uncovered them as clear drivers of the phenomenon. Current employment was not included in any model as it was deemed an intermediate variable. Models eventually excluded the marital status covariate as it presented low variation (less than 10% of the households had a single mother) and was not significantly related to the outcome. This explanation regarding employment was added to the updated "statistical analysis" sub-section.

Comment: *specify the proportion who were married and those who were cohabiting. These are important indicators*
Response: We separated the proportion of "married" and "cohabiting" women and the proportions are now in the text, Tables 1 and 3.

Comment: *what does this mean*
Response: "COV" indicate the Covid visit data point. To avoid confusion we deleted the term from the Table since the data included is already specified in the title of the Table.

Comment: *these variables are missing in the models; provide an explanation*
Response: The variable "having a job last week" was not included in the models as it is most likely an intermediate variable between the "Pandemic" variable and food insecurity. In other words, at least part of the effect of the pandemic is likely to be due to unemployment, therefore adjusting for said variable would cause an underestimation of the true effect. Marital status was initially evaluated but ultimately excluded of the models due to lack of statistical significance, likely due to its low variation. This information has been added to the updated "statistical analysis" sub-section.

Comment: *Include the total wealth quintiles*
Response: The wealth index was categorized into SES quintiles, this is explained in lines 131-148. The value of the index has no intrinsic value, it only indicates if a household is richer than other. That is, the value of the index for each household only ranks households (we know if household "X" is richer than household "Y", but we do not know by how much household "X" is richer than household "Y"). To know "by how much" we would need to use an absolute measure of economic wellbeing such as income or expenditures, but this type of information is not available in our data.
Below we are including the mean value of each SES quintile.

Variable	n	Mean(SD)

**SES (continuous)**	446	0.037(1.51)
**SES (quintiles)**		
1	89	-2.47(1.28)
2	88	-0.22(0.24)
3	88	0.37(0.13)
4	89	0.87(0.16)
5	92	1.58(0.32)

Comment: *be consistent with the unit of analysis; use either women or households throughout the study*
Response: OK, we replace the unit of analyses consistently with “households”.

Comment: *where should Table 2*
Response: We moved Table 2 to place it correctly.

Comment: *this should be T3. In the methods you mentioned that the data was collected between 2020 and 2021. You only have 2020 here*
Response: We added the year “2021” to the heading of Table 2.

Comment: *place the tables at the appropriate location*
Response: We have placed all Tables in the appropriate location.

Comment: *kindly use Mar, Sept, Mar etc instead of mar, sep*
Response: We modified the Figure labels as suggested.

Comment: *did you check whether households that were food insecure in T1 and T2 were the same households that were food insure during COV*
Response: Yes, we checked the transition or change of food insecurity from T2 to the COV stage. Results showed no changes in 52.6% of households (34.5% remained in food security and 18.1% remained in mild food insecurity). 36.1% households changed for the worst (24.2% were food secured in T2 and for the COV stage showed some level of insecurity, and 11.9% changed from mild food insecurity to a more serious levels of insecurity). Finally, 11.3% improved their food insecurity at some level.

Comment: *some of the p’s are caps others are not*
Response: We checked all the p´s and made sure the lower case use is consistent.

Comment: *marital status is missing from this model*
Response: *As explained in the updated "statistical analysis" section, marital status was eventually excluded of all models as it was not significantly related to food insecurity, likely due to its low variation.*

Comment: *month data was collected*

Response: Yes, Figure 1 shows the patterns by month in which the data was collected, which is why it is important to adjust for this variable.

Comment: *delete based on the papers I have showed you*
Response: We deleted the sentence as suggested

Comment: *ref*
Response: We added one new reference to respond a reviewer´s comment: 36. Castillo MJ, Galicia M, Castellano F. Evolución del costo de los alimentos ante el COVID-19. Chile: Centro Latinoamericano para el Desarrollo Rural, 2021 15.

Comment: *considering that 2022 is ending, revise this*
Response: We edited the text as suggested.
