# Supplementary material for: A surge in food insecurity during the COVID-19 pandemic in a cohort in Mexico City
Source: PLoS One. 2024 May 10;19(5):e0297694. doi: 10.1371/journal.pone.0297694 (PMC11086887; doi:10.1371/journal.pone.0297694)
Supplement: Attachment — Submitted filename: Response to reviewers_08Sep2023.pdf [file pone.0297694.s005.pdf]

August 08th 2023

Octavio Gómez-Dantés, MD, MPH
Academic Editor
PLOS ONE

Dear Academic editor.

On behalf of the authors of the original research article **"A surge in food insecurity during the COVID-19 pandemic in a cohort in Mexico City "**, we thank both the Editor and the reviewer´s on the revision of our article. We have worked in the article to address all the comments and suggestions from the reviewer #4, and we are happy to submit a revised version of the manuscript and this rebuttal letter to respond to all comments and suggestions.

We look forward to the revision of this revised version and thank you again for your consideration and time dedicated to our article.

Sincerely,

Martha María Téllez-Rojo, PhD
Corresponding Author

Reviewer #4: 1. *The intro is too long and it is not clear what is the issue under examination. It would be useful to see better structured narrative that is going from the general on food insecurity to the specific issue addressed in this manuscript. Unless you are addressing in a differentiate way the dimensions of food insecurity, there is no need to describe them.*

Response: We have edited the introduction according the reviewer´s comments. We deleted not essential information and the description of the four dimension of food insecurity that a previous reviewer asked to be added. The current version content is as follows. The first paragraph describes the effects of COVID on labor and income, which are major determinants of food insecurity. The second defines food insecurity, and explains how COVID affects food insecurity. The third presents estimates of food insecurity at the beginning of the pandemic as reference. The fourth states the objective of the article.

2. *How you are measuring impact? Your are describen a before and after analysis, that is not an estimation of the impact.*

Response: We understand the reviewer's concern regarding causal language. We have reviewed the use throughout the paper and consider the use of the term "impact" as appropriate. The aim of the study is to quantify the causal effect of the COVID-19 pandemic on food insecurity in the cohort of study. We cannot do a randomized trial to assess this. Our observational data is our only opportunity to estimate this effect. Because of the lack of randomization our results are associations that may not reflect causation, but the aim of our observational study is indeed causal, and we believe the language we use is acceptable. (see Hernán MA. The C-Word: Scientific Euphemisms Do Not Improve Causal Inference From Observational Data. Am J Public Health. 2018)[1]

3. *It is not clear what is the valued added of your study given that there are already data available on the topic in Mexico.*

Response: The main added value of our article is the longitudinal panel data collected at two time points before the pandemic, and one-time point during the COVID pandemic in the same households. This strength is mentioned in the article, describing how most of the literature on the topic comes from cross-sectional analyses. Longitudinal (panel) data that originates from cohort studies, as our article, has many advantages over the, often representative, data that is common in cross-sectional samples. It has been established that panel data, by blending the inter-individual differences and intra-individual dynamics, can provide:

- More accurate inference of model parameters;
- Greater capacity for capturing the complexity of human behavior than a single cross-section or time series data. These include:
    - Constructing and testing more complicated hypotheses
    - Controlling the impact of omitted variables
    - Uncovering dynamic relationships;
    - Generating more accurate predictions for individual outcomes by pooling the data rather than generating predictions of individual outcomes using the data on the individual in question; amongst others.

[1] Hernán MA. The C-Word: Scientific Euphemisms Do Not Improve Causal Inference From Observational Data. Am J Public Health. 2018 May;108(5):616-619. doi: 10.2105/AJPH.2018.304337. Epub 2018 Mar 22.

In summary, we believe the strengths of the longitudinal data in terms of facilitating the finding of causal relationships more than compensates for limitations in terms of representativeness of the sample.

Additionally, we'd like to kindly remind that PLOS ONE's publication criteria focus on the technical and scientific rigor of the work rather than its perceived novelty or broader significance. Our manuscript adheres to these criteria by presenting a methodologically sound study. We believe that every contribution that meets these rigorous scientific standards has value in the scholarly ecosystem, and we are confident that our work meets PLOS ONE's requirements in this regard.

***4.** Additional details on the cohort is required in this paper.*

Response: We added additional details on the cohort.

***5.** Data was collected using different procedures. The period for data collection was not the same for the three data points; also, data was collected in different months of the year. How that could affect the answers? If each of the rounds was collected in a different month and different time frame, it is not clear how your models could correct for that. Please expand the details of your methods to clarify this.*

Response: We thank the reviewer for his/her comments. We do agree that the time of year can have an effect on food insecurity due to potential yearly cyclical changes in the economy. Therefore, we deemed very important to include month of the year as a covariate in the models, to control for this potential confounding effect.

***6.1** The expected value refers to yes/no food insecurity? If that is the case, how it can be above 1?*

Response: We thank the reviewer for raising this important point. The expected value can indeed be above 1 because we are not modeling a binary 'yes/no' outcome for food insecurity. Instead, we are using a food insecurity scale that ranges from 0 to 3. To avoid any confusion, we have clarified this in the statistical analysis section of the revised manuscript.

***6.2** Why the CI of the COVID period is wider?*

Response: We thank the reviewer for her/his insightful question regarding the wider confidence interval (CI) observed during the COVID period. Based on our data, the variability in the food insecurity scale increased during this time, as evidenced by the following:

- Increased Levels of Moderate and Severe Food Insecurity: We observed a significant increase in moderate (from 1.0% in T2 to 9.0% in the COVID period) and severe food insecurity (from 0.6% in T2 to 7.8% in the COVID period).
- Decrease in No Insecurity: The percentage of households with no food insecurity dropped from 58.4% in T2 to 46.2% during the COVID period.
- Mild Food Insecurity: Although mild food insecurity decreased slightly, the pronounced changes in the other categories contribute to the overall increased variability.

This increased variability most likely contributed to the wider CI during the COVID period, as greater variability generally leads to less precise estimates.

6.3 *Which the CI for all periods is wider at the beginning and at the end?*

Response: The wider confidence intervals observed at the beginning and end of each study period are consistent with established statistical principles that are also applicable to local polynomial smoothing methods used in this study. Data points at the extremes have greater 'leverage' and can thus disproportionately influence the model's parameters, leading to increased uncertainty. Additionally, the density of data points is often lower at the extremes, which can compromise the model's ability to make precise estimates. These factors collectively contribute to the wider confidence intervals observed. For a deeper understanding, the reader is referred to 'Applied Linear Statistical Models' by Kutner et al. (2005), which discusses the influence of leverage points and data density on regression estimates, including local polynomial methods.

7. *As your regression is using all observations, it not clear how it can report "protective factors" during the pandemic? Did you interacted those factors with the period?*

Response: The query about reporting 'protective factors' during the pandemic is insightful. In this study, the protective odds ratios are derived from a multiple ordinal regression model that adjusts for various predictors, including study period and socioeconomic status, rather than from interaction terms with the study period. The analysis suggests that these factors are protective against food insecurity in a more general sense, and their protective effect is not specifically tied to the pandemic period. Therefore, it can be reasoned that these factors serve as protective elements against food insecurity regardless of the time frame. Clarifications will be made in the wording of the results section to better articulate this point, specifically, we removed the "during the pandemic" phrase from lines 212-213 in page 13 of the revised version, as that was indeed imprecise.

8. Your discussion should reflect your results only; in your current version, while it acknowledges the lack of generalization, then generalizes.

Response: The reviewer's point about the Discussion section is well-received. While it is our view that acknowledging a study's limitations is not mutually exclusive with discussing its broader implications, we appreciate the concern regarding the perception of generalization. To directly address this, the manuscript's concluding remarks were refined to better articulate the scope of the study's implications. Specifically, the concluding sentence was updated to state: "Our findings contribute to the growing body of evidence suggesting that well-designed and focalized programs are especially crucial during pandemics or natural disasters, to prevent families from the adverse health and well-being effects associated with food insecurity." This adjustment aims to clearly situate our recommendations within the broader context and specify conditions where they are most relevant, thereby addressing concerns about generalization.

***
