# Supplementary material for: A surge in food insecurity during the COVID-19 pandemic in a cohort in Mexico City
Source: PLoS One. 2024 May 10;19(5):e0297694. doi: 10.1371/journal.pone.0297694 (PMC11086887; doi:10.1371/journal.pone.0297694)
Supplement: Attachment — Submitted filename: Review.docx [file pone.0297694.s001.docx]

Review for Article PONE-D-22-12889, Titled, A surge in food insecurity during the COVID-19 pandemic in a cohort in Mexico City

**Summary**

This study’s goal is to estimate the impact of the COVID-19 pandemic on the occurrence of food insecurity in a cohort of women living in Mexico City, and the socioeconomic characteristics associated with food insecurity severity. I can definitely see this paper making contribution to still ongoing issue that countries face due to COVID so it is a very relevant topic. However, the manuscript could be stronger with a few suggestions listed below:

**Abstract**

Typo in the abstract, Results portion

**Study population and sample**

The timings of the visits are difference across the years. Is that a concern? How can that impact results depending on what season of the year/crop cycle it is in?

This should be written differently. “A total of 685 women had data on food security at least one data time point, while 432 women had information on all three time points.”

**Results**

Whereas all other types of food insecurity seem to have increased, it is puzzling that Mild food

insecurity decreased. This is also mentioned the in abstract. More explanation and intuition here could be helpful.

Line 131: missing comma

Table 1: since determinant seems to be pre and post pandemic timing, it would be better to present table 1 by the survey rounds, rather than quintiles (similar to table 2)

Line 177: missing “the”

Figure 1 does not appear where its placed.

**Econometric method**

Usually there are strong reasons behind why authors would choose to use FE versus RE models. In this paper, there is no justification or mention of why both are done? Also, there are no robustness checks of the models.

Since the month of data collection is different, I thought I saw somewhere that authors control for that, however, I don’t see that in Table 4. And how does controlling for months correlate with the survey rounds themselves? What is the correlation?

The authors state that they used a host of characteristics but they only present the ones that are statistically significant. Are the rest of the variables not presented because authors are running out of space in the paper? Mention of these variables in table notes would at least be expected so readers can see it at a glance.

**Discussion**

“To our knowledge, this is the first study with panel data that estimated the magnitude of the COVID-19 impact on food insecurity in Mexico”. I might be wrong but I thought that the World Bank did it for many countries across the world and that should have included Mexico.

Line 217: grammar

Line 267: grammar

Line 269: the definition should be written early on since the study is about food insecurity
